# Molecular mapping of QTLs for grain dimension traits in Basmati rice

**DOI:** 10.3389/fgene.2022.932166

**Published:** 2022-08-02

**Authors:** Ankit Malik, Aruna Kumar, Ranjith Kumar Ellur, Gopala Krishnan S, Deepshikha Dixit, Haritha Bollinedi, KK Vinod, M Nagarajan, PK Bhowmick, NK Singh, AK Singh

**Affiliations:** ^1^ Division of Genetics, ICAR-Indian Agricultural Research Institute (ICAR-IARI), New Delhi, India; ^2^ Amity Institute of Biotechnology, Amity University, Noida, India; ^3^ Rice Breeding and Genetics Research Centre, ICAR-IARI, Aduthurai, India; ^4^ ICAR-National Institute for Plant Biotechnology, IARI, New Delhi, India

**Keywords:** quantitative trait loci, basmati rice, grain quality, SSR markers, mapping

## Abstract

Basmati rice is known for its extra-long slender grains, exceptional kernel dimensions after cooking, high volume expansion, and strong aroma. Developing high yielding Basmati rice varieties with good cooking quality is a gigantic task. Therefore, identifying the genomic regions governing the grain and cooked kernel dimension traits is of utmost importance for its use in marker-assisted breeding. Although several QTLs governing grain dimension traits have been reported, limited attempts have been made to map QTLs for grain and cooked kernel dimension traits of Basmati rice. In the current study, a population of recombinant inbred lines (RIL) was generated from a cross of Sonasal and Pusa Basmati 1121 (PB1121). In the RIL population, there was a significant positive correlation among the length (RRL: rough rice length, MRL: milled rice length, CKL: cooked kernel length) and breadth (RRB: rough rice breadth, MRB: milled rice breadth and CKB: cooked kernel breadth) of the related traits, while there was significant negative correlation between them. QTL mapping has led to the identification of four major genomic regions governing MRL and CKL. Two QTLs co-localize with the earlier reported major gene *GS3* and a QTL *qGRL7.1,* while the remaining two QTLs *viz., qCKL3.2* (*qMRL3.2*) and *qCKL4.1* (*qMRL4.1*) were novel. The QTL *qCKL3.2* has been bracketed to a genomic region of 0.78 Mb between the markers RM15247 and RM15281. Annotation of this region identified 18 gene models, of which the genes predicted to encode pentatricopeptides and brassinosteroid insensitive 1-associated receptor kinase 1 precursor may be the putative candidate genes. Furthermore, we identified a novel QTL *qKER2.1* governing kernel elongation ratio (KER) in Basmati rice.

## Introduction

Basmati rice attracts consumers worldwide due to its pleasant aroma and unique grain and cooking quality parameters. Among the Basmati rice varieties developed, PB1121 stands out due to its exclusive grain qualities, making it one of the most traded crop varieties in the world. It possesses extra-long slender grains, very long cooked kernel (upto 22 mm), high kernel elongation ratio, volume expansion of more than four times upon cooking, and pleasant aroma. The grain dimension traits that determine rice grain quality are the length and breadth of rough, milled, and cooked rice grain as well as the kernel elongation ratio (KER). The genetics of rice grain size and weight has been extensively studied and >300 QTLs have been mapped (www.gramene.org) (Bhatia et al., 2018). However, only few of them *viz., GS3* (Fan et al., 2006), *GS5* (Li et al., 2011), *GS7* (Shao et al., 2012), *qGL3* (Zhang et al., 2012b), *GW2* (Song et al., 2007), *GW8* (Wang et al., 2012), *TGW6* (Ishimaru et al., 2013), *GW5* (Duan et al., 2017), *qTGW3* (Zejun et al., 2018), and *qGS10* (Zhu et al., 2019) have been cloned and functionally validated. Among these, *GS3* has been identified as a major gene governing the rice grain length and weight (Mao et al., 2010). Several polymorphic loci of *GS3 viz,* SR_17_ (2nd intron), RGS_1_ (4th intron), RGS_2_ (5th exon), *aksGS3-12* (5th exon) etc., have been reported (Wang et al., 2011; Anand et al., 2013a). The gene *qGL3* has been identified to encode a putative serine/threonine protein phosphatase that contains a Kelch-like repeat domain and acts on its cell cycle-related substrate, Cyclin-T1; 3, to increase the rice grain length (Zhang et al., 2012b).

Several independent studies have identified QTLs for the grain width and weight. In a large-grained genotype WY3, a major gene *GW2* encoding a RING-type E3 ubiquitin ligase was identified wherein, a single base pair deletion results into premature stop codon, thereby reducing its expression while increasing the grain width and weight (Song et al., 2007). *GW5* was mapped on chromosome 5 which encodes a putative serine carboxypeptidase, wherein a deletion increases the cell number in the outer glume of the rice flower thereby regulating the grain width, filling, and weight (Li et al., 2011). *TGW6* was mapped on chromosome 6 which encodes a novel protein with an indole-3-acetic acid (IAA)-glucose hydrolase activity and its loss of function enhances the rice grain weight (Ishimaru et al., 2013).

Identifying genomic regions governing grain dimension traits in Basmati rice genotypes helps in their precise manifestation in Basmati improvement programs. Earlier, in a RIL population generated from a cross Pusa 1342/PB1121, several novel QTLs governing grain length, elongation ratio, and aroma in Basmati rice were identified (Amarawathi et al., 2008). Subsequently, a major gene *GW8* promoting cell division and proliferation thereby enhancing the grain width and yield was identified in a traditional Basmati rice variety, Basmati 385 (Wang et al., 2012). Furthermore, three QTLs for grain elongation on chromosomes 4 (*qGE4.1*) and 6 (*qGE6.1* and *qGE6.2*) were identified in a Basmati rice genotype (Arikit et al., 2019).

Although, Basmati rice possesses unique grain dimension traits such as extra-long slender grains and exceptional cooked kernel dimensions, there are limited efforts made toward identifying the genomic regions governing these traits. In the present study, we attempted to map QTLs governing the grain and cooked kernel dimension traits using a RIL population generated from the cross of Sonasal and PB1121.

## Materials and methods

### Development of the mapping population

**FIGURE 1:**
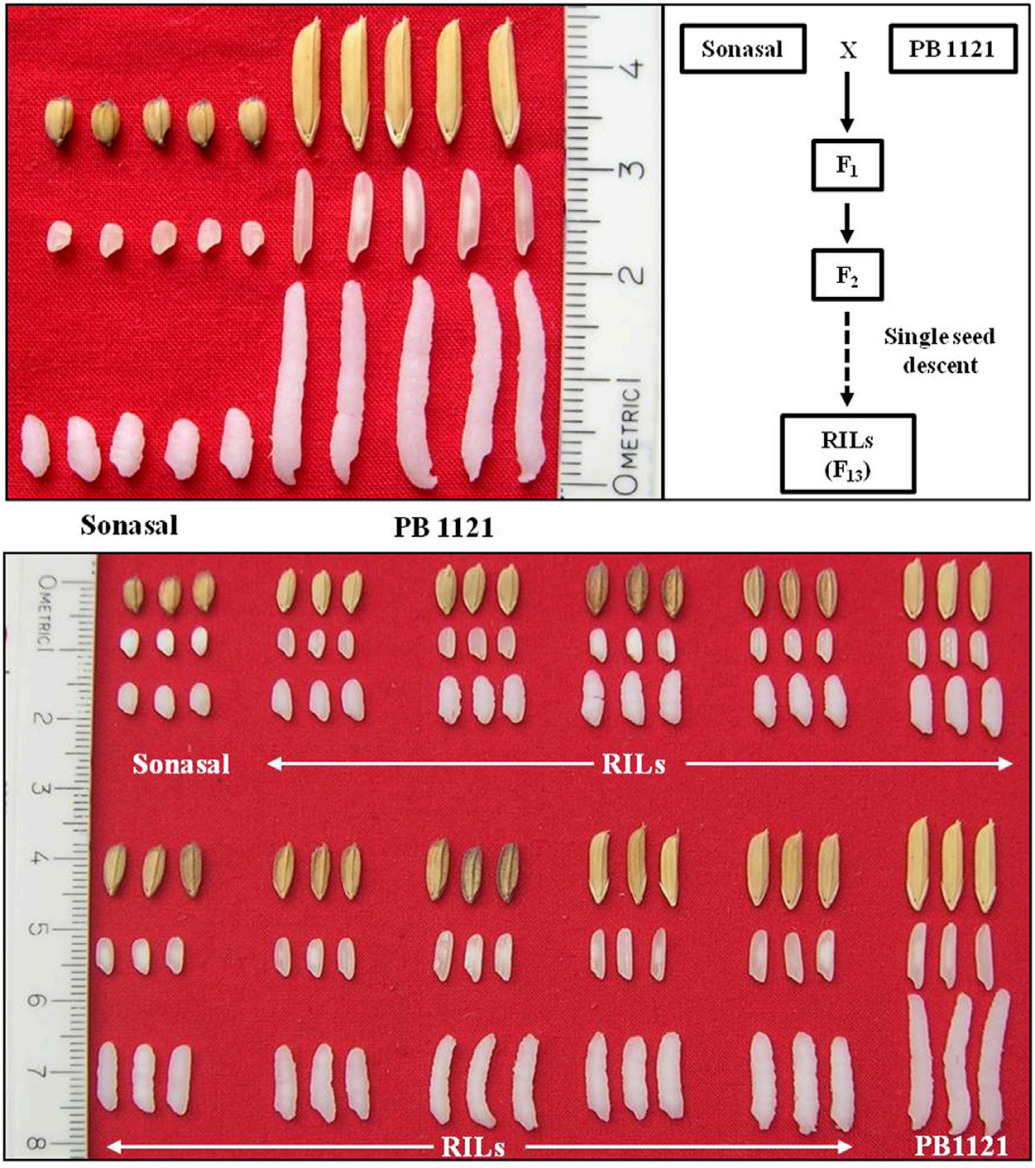
Variation for grain dimension traits between parental lines, Sonasal, and PB1121; and RIL population.

A RIL population comprising of 173 lines was developed by crossing an aromatic short grain genotype Sonasal with an extra-long grained Basmati rice variety PB1121. Sonasal has extremely shorter grains, which are at least 50% shorter than that of PB1121, with slightly wider grains. Both parents were aromatic. The population was advanced till the F_13_ generation through the single seed descent method (Figure 1).

### Evaluation of grain dimension traits

The RIL population along with the parental lines and checks were grown in an augmented randomized block design following all the standard agronomic practices. The seed harvested from each of the lines was shade-dried to a uniform moisture content of 14% and stored at room temperature. Observations on rough, milled, and cooked grain dimension traits were recorded after six months. The grain dimension traits namely, RRL, RRB, MRL, MRB, CKL, and CKB were recorded in three replications with ten grains in each replication. For CKL and CKB, ten milled rice grains were soaked in 20 ml of distilled water for 20 min in a test tube. The samples were placed in a water bath at 100°C for 7 min followed by cooling them to room temperature. A photo analyzer was used (C-DAC) to record the data on the length and breadth of the grains.

### Genotyping and data analysis

DNA was extracted using a standard CTAB (Cetyl- Tri Methyl Ammonium Bromide) method (Murray and Thompson, 1980). The PCR reaction was carried out using 25 ng of genomic DNA, 1X PCR assay buffer with 1.5 mM MgCl2, 5 pmol of forward and reverse primers, 0.05 mM dNTPs, and 1U of Taq polymerase. The final reaction volume was made up to 10 μl using nuclease-free water. Amplified PCR products were resolved by 3.5% agarose gel electrophoresis and documented on a gel documentation system (BIO-RAD Gel Doc XR+, California, United States). A total of 1,054 markers comprising of sequence-tagged microsatellite site (STMS) and gene-based markers were used in a parental polymorphism survey. The polymorphic markers were used to genotype the RIL population. The linkage map was constructed using the QTL IciMapping software (Li et al., 2007; Meng et al., 2015). Grouping and ordering of polymorphic markers were carried out using the regression mapping algorithm using recombination frequency (REC) with a default value set to 0.3. Furthermore, rippling was carried out for fine-tuning the ordered markers on their respective chromosomes using the REC algorithm with a default window size of 5. QTLs were identified using the inclusive composite interval mapping (ICIM) algorithm for additive gene effects.

## Results

### Phenotyping of the RIL population

**FIGURE 2:**
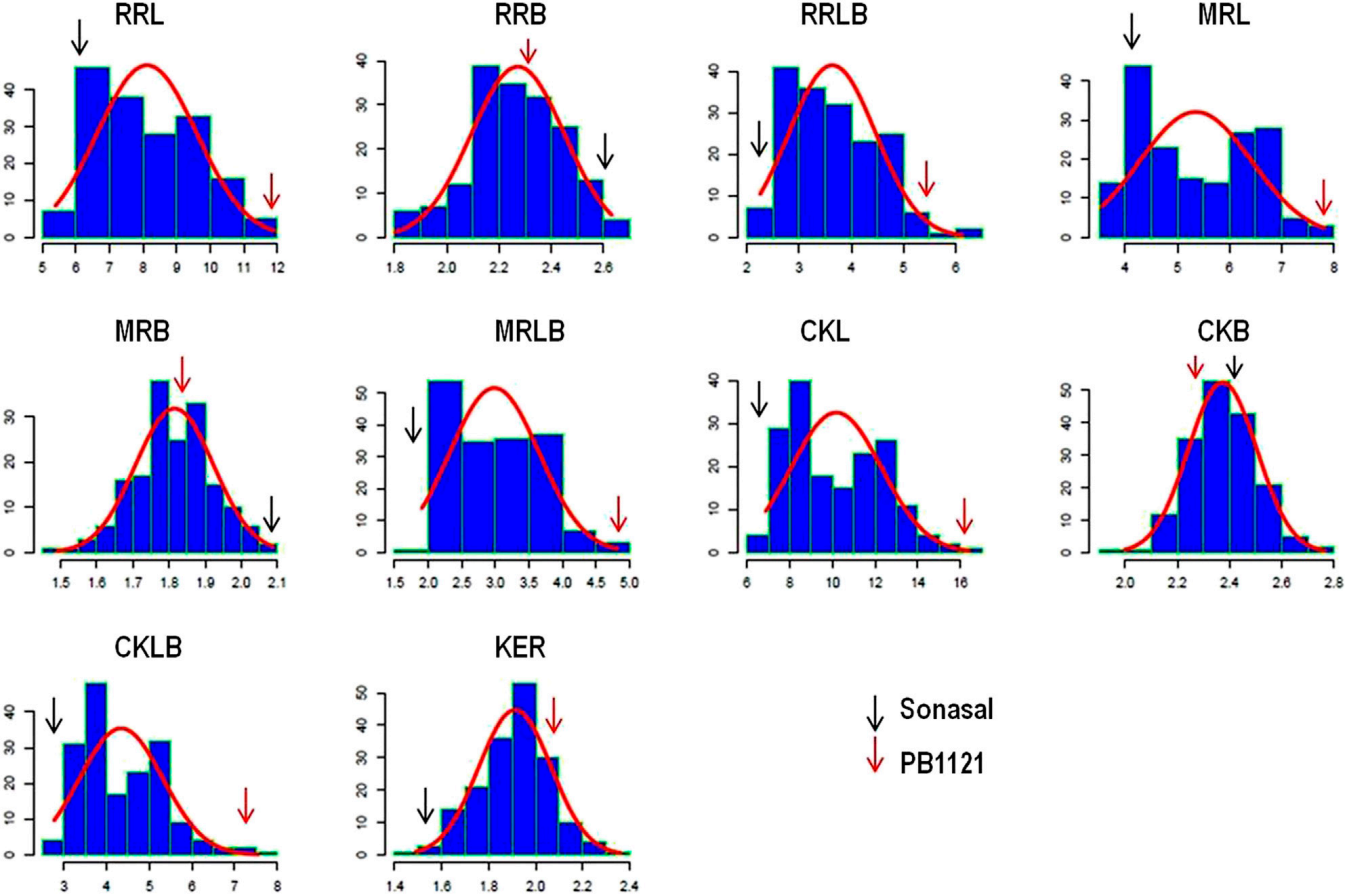
Distribution pattern of grain dimension traits in an RIL population. rough rice length (RRL), rough rice breadth (RRB), rough rice length to breadth ratio (RRLB), milled rice length (MRL), milled rice breadth (MRB), milled rice length to breadth ratio (MRLB), cooked kernel length (CKL), cooked kernel breadth (CKB), cooked kernel length to breadth ratio (CKLB), and kernel elongation ratio (KER).

The RIL population comprising of 173 lines were evaluated during three subsequent crop growing seasons, *viz., Kharif* 2019, 2020, and 2021 (Table 1). The population showed significant variations for all the grain dimension-related traits. The RRL ranged from 4.67 to 12.33 mm while, RRB ranged from 1.40 to 3.00 mm. The corresponding measures for RRL in Pusa Basmati 1121 were 12.43 and 6.37 mm in Sonasal. Similarly, for RRB, the parents showed average values of 2.30 and 2.60 mm for Pusa Basmati 1121 and Sonasal, respectively. The MRL among the RILs varied from 3.37 to 8.07 mm, while Pusa Basmati 1121 measured 8.07 mm and Sonasal 4.07 mm. For MRB, RILs ranged between 1.33 and 2.47 mm, while the parents recorded values of 1.80 and 2.20 mm, respectively for Pusa Basmati 1121 and Sonasal. There was a huge variation for CKL which ranged from 6.27 to 17.13 mm among the RILs, while CKB ranged between 1.78 and 3.10 mm. CKL and CKB for Pusa Basmati 1121 were 16.60 and 2.33 mm, respectively, while that of Sonasal was 6.25 and 2.47 mm, in that order. Among the RILs, KER varied from 1.23 to 2.59, compared to 2.06 and 1.54 recorded in Pusa Basmati 1121 and Sonasal, respectively. The phenotypic coefficient of variation (PCV) among the traits ranged from a minimum of 7.12% for CKB to a maximum of 25.86% for MRLB during *Kharif* 2019, while during *Kharif* 2020, it ranged from 6.40% for CKB to 24.83% in RRLB. During *Kharif* 2021, PCV was minimum for MRB (5.14%) and maximum for RRLB (23.67%). The PCV for KER ratio was 9.33%, 10.83%, and 10.84% during *Kharif* 2019, *Kharif* 2020, and *Kharif* 2021, respectively (Table 1; Figure 1). The transgressive segregation was observed for the traits RRL, MRL, RRB, MRB, CKB, and KER (Figure 2).

**TABLE 1:** Descriptive statistics of Sonasal, PB1121, and RIL population.

Traits	Sonasal	PB1121	Variability in RILs *Kharif* 2019			Variability in RILs *Kharif* 2020			Variability in RILs *Kharif* 2021		
			Mean ± SE	Range (mm)	CV	Mean ± SE	Range (mm)	CV	Mean ± SE	Range (mm)	CV
RRL	6.37	12.43	8.35 ± 0.12	5.67–12.33	19.29	8.35 ± 0.11	5.80–12.07	17.17	7.64 ± 0.12	4.67–11.37	19.87
RRB	2.60	2.30	2.40 ± 0.02	1.87–3.00	8.49	2.39 ± 0.02	1.73–2.93	10.69	2.02 ± 0.01	1.40–2.67	9.13
RRLB	2.45	5.40	3.52 ± 0.06	2.28–6.54	23.44	3.56 ± 0.07	2.28–6.85	24.83	3.82 ± 0.07	2.03–6.24	23.67
MRL	4.07	8.07	5.31 ± 0.08	3.47–7.73	20.94	5.33 ± 0.08	3.53–7.73	19.68	5.42 ± 0.09	3.37–8.07	21.57
MRB	2.20	1.80	1.88 ± 0.01	1.40–2.47	9.19	1.90 ± 0.01	1.47–2.33	7.43	1.66 ± 0.01	1.33–2.00	5.14
MRLB	1.85	4.48	2.86 ± 0.06	1.80–4.76	25.43	2.83 ± 0.05	1.76–4.78	22.62	3.27 ± 0.06	1.94–5.63	22.42
CKL	6.25	16.60	10.23 ± 0.18	6.27–17.13	23.41	10.18 ± 0.16	6.80–16.20	20.27	10.15 ± 0.16	6.83–15.90	20.87
CKB	2.47	2.33	2.47 ± 0.01	2.07–2.87	7.12	2.48 ± 0.01	2.00–2.93	6.40	2.17 ± 0.02	1.78–3.10	9.35
CKLB	2.53	7.11	4.17 ± 0.08	2.64–7.56	25.86	4.13 ± 0.07	2.52–7.55	22.44	4.72 ± 0.08	2.64–7.81	23.26
KER	1.54	2.06	1.93 ± 0.01	1.29–2.25	9.33	1.92 ± 0.02	1.23–2.59	10.83	1.89 ± 0.02	1.37–2.58	10.84

SE, standard error; CV, coefficient of variance; RRL, rough rice length; RRB, rough rice breadth; RRLB, rough rice length to breadth ratio; MRL, milled rice length; MRB, milled rice breadth; MRLB, milled rice length to breadth ratio; CKL, cooked kernel length; CKB, cooked kernel breadth; CKLB, cooked kernel length to breadth ratio; KER, kernel elongation ratio.

### Correlation between the grain dimension traits in the RIL population

**FIGURE 3:**
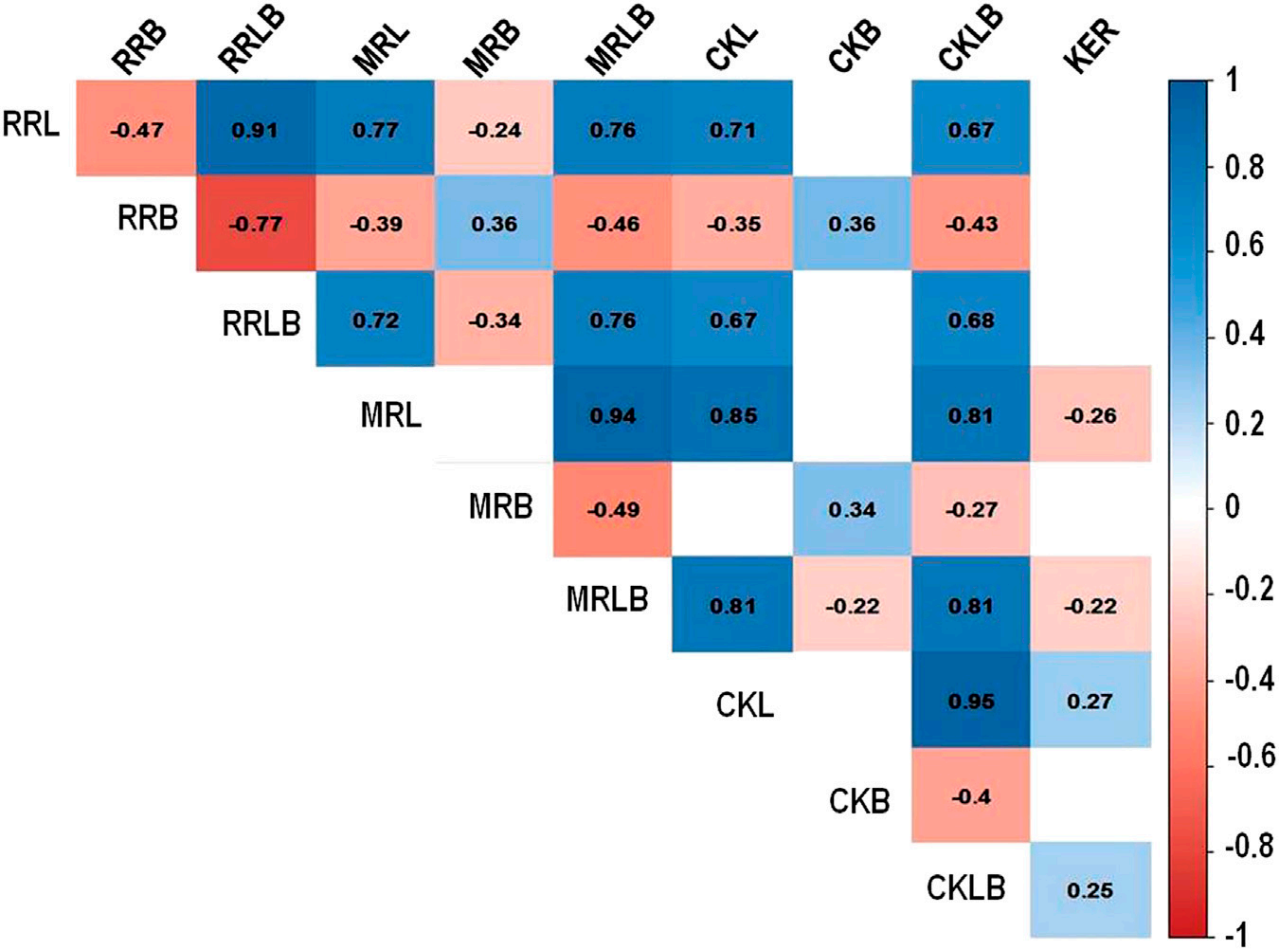
Correlation among grain dimension traits in rice. Rough rice length (RRL), rough rice breadth (RRB), rough rice length to breadth ratio (RRLB), milled rice length (MRL), milled rice breadth (MRB), milled rice length to breadth ratio (MRLB), cooked kernel length (CKL), cooked kernel breadth (CKB), cooked kernel length to breadth ratio (CKLB), and kernel elongation ratio (KER).

The significant positive correlation was observed among the grain length and length/breadth (rough rice, milled, and cooked) traits. However, there was a significant negative correlation between the grain length and breadth traits. Furthermore, there was a significant positive correlation between CKL and KER; while negative correlation was observed between CKB and CKL, MRLB, and KER; and no correlation between KER and rough rice dimension traits (Figure 3).

### Construction of a genetic linkage map

A total of 116 markers polymorphic between Sonasal and PB1121 were used to construct the linkage map. A total of 12 linkage groups were constructed with a cumulative map distance of 2,469.38 cM. The number of markers on different chromosomes ranged from 4 on chromosome 10 to 16 on chromosome 3 (Table 2). The average genetic distance between two adjacent markers was 21.29 cM.

**TABLE 2:** Chromosome-wise number of the markers used for construction of linkage map, map length, and average marker interval.

Chromosome	No. of markers	Map length (cM)
1	15	325.04
2	11	287.46
3	16	349.74
4	8	183.98
5	11	192.93
6	8	189.63
7	11	165.21
8	10	160.65
9	6	159.70
10	4	136.33
11	9	164.54
12	7	154.17
**TOTAL**	**116**	**2469.38**

cM, centi Morgan.

### QTLs mapped

**FIGURE 4:**
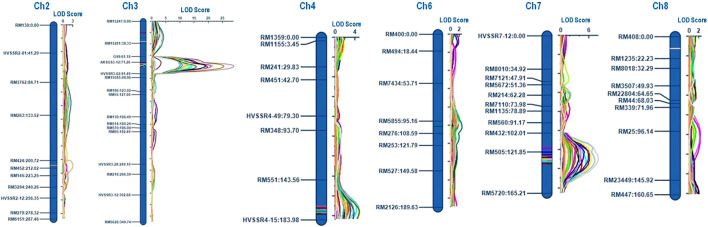
Graphical representation of identified QTLs on chromosomes.

QTLs were identified for ten grain dimension traits using ICIM. A total of twenty-three QTLs were detected on six different chromosomes (chromosomes 2, 3, 4, 6, 7, and 8) for grain dimension-related traits (Figure 4). For each of these grain dimension traits, the proportion of phenotypic variance explained (PVE), its additive effect, nearest left marker, and nearest right marker were determined (Table 3).

**TABLE 3:** QTLs identified for grain quality parameters by ICIM for three seasons.

QTL Name	Trait name	Chr	Position	Left marker	Right marker	LOD	PVE (%)	Add
*qRRL3.1*	RRL_2019	3	79	aksGS3-12	HvSSR03-62	19.67	65.92	1.30
	RRL_2020	3	79	aksGS3-12	HvSSR03-62	15.74	62.57	1.13
	RRL_2021	3	79	aksGS3-12	HvSSR03-62	13.53	51.37	1.09
*qRRL4.1*	RRL_2019	4	172	RM551	HvSSR04-15	2.59	10.86	0.53
	RRL_2021	4	174	RM551	HvSSR04-15	4.35	18.11	0.65
*qRRL7.1*	RRL_2019	7	134	RM505	RM5720	4.47	20.86	0.74
	RRL_2020	7	134	RM505	RM5720	3.89	19.19	0.63
	RRL_2021	7	132	RM505	RM5720	5.35	21.95	0.71
*qRRB3.1*	RRB_2020	3	72	aksGS3-12	HvSSR03-62	3.37	7.96	−0.07
*qRRB7.1*	RRB_2019	7	122	RM505	RM5720	4.95	12.49	−0.07
	RRB_2020	7	128	RM505	RM5720	6.04	23.48	−0.12
*qRRLB3.1*	RRLB_2019	3	78	aksGS3-12	HvSSR03-62	12.67	46.15	0.56
	RRLB_2020	3	77	aksGS3-12	HvSSR03-62	10.17	33.10	0.51
	RRLB_2021	3	77	aksGS3-12	HvSSR03-62	9.79	30.11	0.50
*qRRLB4.1*	RRLB_2020	4	176	RM551	HvSSR04-15	3.01	10.03	0.28
	RRLB_2021	4	174	RM551	HvSSR04-15	4.88	20.51	0.41
*qRRLB7.1*	RRLB_2019	7	129	RM505	RM5720	6.45	22.41	0.39
	RRLB_2020	7	129	RM505	RM5720	7.92	26.35	0.45
	RRLB_2021	7	131	RM505	RM5720	7.23	29.21	0.49
*qMRL3.1*	MRL_2019	3	78	aksGS3-12	HvSSR03-62	25.46	64.59	0.89
	MRL_2020	3	78	aksGS3-12	HvSSR03-62	20.91	76.14	0.91
	MRL_2021	3	78	aksGS3-12	HvSSR03-62	28.91	75.67	1.01
*qMRL3.2*	MRL_2020	3	20	RM15247	RM15281	2.95	12.92	0.38
*qMRL4.1*	MRL_2019	4	177	RM551	HvSSR04-15	4.90	14.34	0.42
	MRL_2021	4	170	RM551	HvSSR04-15	2.98	13.65	0.43
*qMRL7.1*	MRL_2019	7	127	RM505	RM5720	3.13	7.36	0.30
	MRL_2021	7	131	RM505	RM5720	2.58	8.72	0.34
*qMRB7.1*	MRB_2019	7	126	RM505	RM5720	4.27	15.54	−0.07
	MRB_2020	7	118	RM432	RM505	3.14	10.86	−0.05
*qMRLB3.1*	MRLB_2019	3	78	aksGS3-12	HvSSR03-62	18.90	54.77	0.54
	MRLB_2020	3	78	aksGS3-12	HvSSR03-62	19.20	66.60	0.52
	MRLB_2021	3	77	aksGS3-12	HvSSR03-62	21.56	58.28	0.56
*qMRLB4.1*	MRLB_2019	4	178	RM551	HvSSR04-15	3.72	9.83	0.23
	MRLB_2021	4	174	RM551	HvSSR04-15	3.70	14.30	0.28
*qMRLB7.1*	MRLB_2019	7	127	RM505	RM5720	5.86	14.52	0.28
	MRLB_2020	7	125	RM505	RM5720	3.85	9.04	0.19
	MRLB_2021	7	130	RM505	RM5720	4.07	12.11	0.25
*qCKL3.1*	CKL_2019	3	75	aksGS3-12	HvSSR03-62	10.15	24.49	1.18
	CKL_2020	3	77	aksGS3-12	HvSSR03-62	10.78	29.81	1.12
	CKL_2021	3	79	aksGS3-12	HvSSR03-62	14.22	58.26	1.62
*qCKL3.2*	CKL_2019	3	27	RM15247	RM15281	3.88	13.01	0.86
	CKL_2020	3	28	RM15247	RM15281	4.27	13.96	0.77
*qCKL4.1*	CKL_2019	4	177	RM551	HvSSR04-15	3.83	11.89	0.83
	CKL_2020	4	175	RM551	HvSSR04-15	3.11	11.26	0.70
*qCKL6.1*	CKL_2020	6	101	RM5855	RM276	2.62	5.60	0.49
*qCKLB3.1*	CKLB_2019	3	73	aksGS3-12	HvSSR03-62	12.82	32.51	0.61
	CKLB_2020	3	75	aksGS3-12	HvSSR03-62	9.41	23.59	0.45
	CKLB_2021	3	77	aksGS3-12	HvSSR03-62	11.51	37.27	0.67
*qCKLB3.2*	CKLB_2020	3	35	RM15247	RM15281	3.32	7.55	0.25
*qKER2.1*	KER_2020	2	208	RM424	RM452	3.05	9.56	0.07

Chr, chromosome; LOD, logarithm of the odds; PVE, phenotypic variance; Add, additive effect; RRL, rough rice length; RRB, rough rice breadth; RRLB, rough rice length to breadth ratio; MRL, milled rice length; MRB, milled rice breadth; MRLB, milled rice length to breadth ratio; CKL, cooked kernel length; CKB, cooked kernel breadth; CKLB, cooked kernel length to breadth ratio; KER, kernel elongation ratio.

### QTL for rough rice length and breadth

A total of eight QTLs associated with rough rice dimension traits were identified on chromosomes 3, 4, and 7 across 3 years of testing. A novel QTL *qRRL4.1* flanking the markers RM551 and HvSSR04-15 was identified on chromosome 4 with PVE of 10.86% and 18.11% during the years 2019 and 2021, respectively. The PVE of *qRRL3.1* ranged from 51.37% to 65.92% and the PVE of *qRRL7.1* ranged from 19.19% to 21.95% during 3 years of testing. The QTLs *qRRL3.1* and *qRRL7.1* correspond to already cloned genes *GS3* and *qGRL7.1,* respectively.

The trait RRB was found to mainly governed by two major QTLs *qRRB3.1* (PVE: 3.37%) and *qRRB7.1* (PVE: 12.49% and 23.48%) and for the trait RRLB, two robust QTLs *qRRLB3.1* (PVE: 30.11–46.15%) and *qRRLB7.1* (PVE: 22.41–29.21%) were mapped on chromosome 3 and chromosome 7 which correspond to the major genes *GS3* and *qGRL7.1*, respectively (Table 3). A major novel QTL *qRRLB4.1* flanking the region RM551 and HvSSR04-15 was identified for the trait RRLB on chromosome 4 during *Kharif* 2020 and *Kharif* 2021 which explained the phenotypic variance of 10.03%–20.51%, respectively.

### QTLs for milled rice length and breadth

A total of four QTLs were found to govern the trait MRL. The major QTL *qMRL3.1* was identified on chromosome 3 with PVE of 64.59%–76.14% during 3 years of testing. The QTL *qMRL7.1* was mapped on chromosome 7 which accounted for the PVE of 7.36 and 8.72% during *Kharif* 2019 and *Kharif* 2020, respectively. Two novel QTLs, namely, *qMRL3.2* flanked by the markers RM15247 and RM15281; and *qMRL4.1* flanked by the markers RM551 and HvSSR04-15 located on chromosomes 3 and 4, respectively, were mapped. The PVE of QTL *qMRL3.1* was 12.92% during *Kharif* 2020 while, the PVE of *qMRL4.1* was 14.34% and 13.65% during *Kharif 2019* and *Kharif 2021,* respectively.

For the trait MRB, a robust QTL *qMRB7.1* was identified on chromosome 7 with PVE of 15.54% and 10.86% during *Kharif* 2019 and *Kharif* 2020, respectively. For the trait MRLB, two robust QTLs, namely, *qMRLB3.1* and *qMRLB7.1* were mapped on chromosomes 3 and 7, respectively. Another novel QTL *qMRLB4.1* flanked by the markers RM551 and HvSSR04-15 with PVEs of 9.83% and 14.30% during *kharif* 2019 and *kharif* 2020, respectively.

### QTLs for cooked kernel length and breadth

CKL is one of the key Basmati traits which determine the cooking quality. In the current population, four QTLs governing the trait CKL were identified of which three QTLs namely; *qCKL3.2, qCKL4.1,* and *qCKL6.1* were identified to be novel. A major QTL *qCKL3.1* with PVE ranging from 29.81% to 58.26% and an additive effect ranging from 1.12 to 1.62 mm was identified during all the 3 years of testing. The QTL *qCKL3.2* (PVE: 13.01%–13.96%) flanked by RM15247 and RM15281 and the QTL *qCKL4.1* (PVE: 11.26%–11.89%) flanked by the markers RM551 and HvSSR04-15 were identified during *Kharif* 2019 and *Kharif* 2020. Another novel QTL, *qCKL6.1* was mapped on chromosome 6 with PVE of 5.60% during *Kharif* 2020. The promising QTL *qCKL3.2* was bracketed between the markers RM15247 and RM15281 which spans a region of 0.78 Mb (17.90 Mb–18.68 Mb) on chromosome 3. The region comprises of 114 annotated gene models, of which 18 genes were putative candidates which may play a role in determining the grain length (Table 4).

**TABLE 4:** The predicted gene models in the novel QTLs qCKL3.2/qMRL3.2

Gene models	Start position (bp)	Stop position (bp)	Putative function
LOC_Os03g31480	17934776	17935790	expansin precursor, putative, expressed
LOC_Os03g31550	17985563	17998498	aldehyde oxidase, putative, expressed
LOC_Os03g31594	18026740	18041873	jmjC domain containing protein, expressed
LOC_Os03g31679	18085558	18087371	annexin A7, putative, expressed
LOC_Os03g31690	18087460	18093916	GCN5-related N-acetyltransferase, putative, expressed
LOC_Os03g32030	18318940	18319467	DNA-directed RNA polymerase II subunit RPB1, putative, expressed
LOC_Os03g32050	18325230	18332522	peroxidase precursor, putative, expressed
LOC_Os03g32090	18355904	18359839	pentatricopeptide, putative, expressed
LOC_Os03g32160	18397036	18401598	calmodulin binding protein, putative, expressed
LOC_Os03g32180	18410063	18411482	polygalacturonase inhibitor 1 precursor, putative, expressed
LOC_Os03g32220	18430111	18431229	ZOS3-11 - C2H2 zinc finger protein, expressed
LOC_Os03g32230	18435989	18437090	ZOS3-12 - C2H2 zinc finger protein, expressed
LOC_Os03g32270	18450996	18456312	hydrolase, alpha-beta fold family domain containing protein, expressed
LOC_Os03g32314	18485606	18488371	allene oxide cyclase 4, chloroplast precursor, putative, expressed
LOC_Os03g32580	18635876	18638518	BRASSINOSTEROID INSENSITIVE 1-associated receptor kinase 1 precursor, putative, expressed
LOC_Os03g32590	18638520	18647607	transcription initiation factor, putative, expressed
LOC_Os03g32620	18677824	18682844	pentatricopeptide, putative, expressed
LOC_Os03g32630	18686894	18689388	ABC transporter, ATP-binding protein, putative, expressed

### QTLs for kernel elongation ratio

KER is one of the key traits of Basmati rice which determines the extent of the linear elongation of the grain upon cooking. A novel QTL *qKER2.1* was mapped on chromosome 2 flanked by markers RM424 and RM452 with PVE of 9.56% during *Kharif* 2020.

## Discussion

Rice grain shape is mainly determined by the grain length and breadth. These traits are under the influence of a few large effects and multiple small effect genes, thus possessing complex patterns of inheritance. All the grain dimension traits showed a high degree of phenotypic variation in the RIL population during all the 3 years of testing. Furthermore, the grain dimension traits showed a near-normal distribution with slight skewness toward either of the parental lines which indicated the influence of the combination of few oligo-genes and many minor genes in determining these traits. The significant positive correlation was observed among length-related traits while a significant negative correlation was observed between the length- and breadth-related traits. The observed correlation among the traits in the present study was consistent with the previous studies (Amarawathi et al., 2008; Bai et al., 2010).

The linkage map comprising of 12 linkage groups with a cumulative map distance of 2,469.38 cM was constructed using 116 polymorphic markers with an average marker interval distance of 21.29 cM. Although the average marker interval distance was 21.29 cM, the genetic distances between neighboring markers varied across the linkage groups due to the spatial variation in recombination frequencies. Sonasal is a short grain aromatic rice variety and PB1121 is an extra-long grain Basmati rice variety with sufficient genomic and phenotypic diversity among the parental lines. It was demonstrated that there exists tremendous diversity between the aromatic short grain and Basmati varieties (Garris et al., 2005), indicating the existence of tremendous structural changes, which may lead to the occurrence of segregation distortion at a certain loci in the mapping population generated. There are several possible reasons for segregation distortion, such as gametic or zygotic selection, genetic incompatibility, chromosome rearrangement, pollen competition, preferential fertilization etc., (Xu et al., 1997).

The genetic and molecular dissection of grain dimension parameters in rice have received a lot of attention, and until now, several QTLs have been identified and few genes have been functionally characterized (www.gramene.org). The major focus has been to understand the genetics and the identification of genomic regions governing the grain length and grain width in non-Basmati rice genotypes. This has resulted in the identification of major effect QTLs/genes such as *GS3*, a major gene governing the grain length and weight which was identified in a population generated from a cross Minghui 63/Chaun 7; *GW2*, a major gene for the grain width and weight mapped in a population generated from a cross WY3/Fengaizhan 1; *GS7*, associated with grain length was mapped using a population generated from a cross D50/HB277; *qSW5*, associated with the grain breadth was mapped in a population Nipponbare/Kasalath etc.,

Basmati is a unique combination of grain and cooking quality parameters with a strong and pleasing aroma. However, limited attempts were made to understand the grain and cooked rice dimension-related traits in Basmati genotypes. Earlier, in a population generated from the cross Basmati 385 and HJX74, a major gene *GW8* was identified. In the present study, a RIL population was generated using highly contrasting parental lines for the target traits. The major effect QTLs *viz., qRRL3.1, qRRLB3.1, qMRL3.1, qMRLB3.1,* and *qCKL3.1* were found to be co-mapped to a genomic region on chromosome 3 which is known to possess the *GS3* gene. The functional allele of *GS3* reduces the grain size and weight (Fan et al., 2009). In *GS3* a C to A mutation in the exon 2 introduces a stop codon leading to long grains. Based on this mutation, a CAPS marker SF28 was developed for its use in molecular breeding (Fan et al., 2009). Furthermore, previously we identified a novel indel *aksGS3-12* in the short grain genotype Sonasal, which was predominant in short grain landraces (Anand et al., 2013b). The functional allele at SF28 reduces the grain size, and if coupled with the 342bp deletion at *aksGS3-12*, it may further reduce the grain size, as in Sonasal. In the current study, it has been demonstrated that *GS3* has a key role in governing rough, milled, and cooked rice length as well as MRLB. The other major effect QTLs identified were *qRRL7.1, qRRB7.1, qMRL7.1, qMRB7.1,* and *qMRLB7.1* which were co-mapped to the genomic region on chromosome 7. This region co-localizes with the earlier reported QTL *qGRL7.1* (Amarawathi et al., 2008).

The novel QTLs *viz., qMRL3.2*, *qCKL3.2,* and *qCKLB3.2* are co-mapped between the markers RM15247 and RM15281 on chromosome 3. The annotation of the QTL-bracketed region of 0.78 Mb identified 18 putative candidate gene models which may have a significant role in determining the kernel length. This genomic region possessed gene models which code for pentatricopeptides, brassinosteroid insensitive 1-associated receptor kinase 1 precursor and transcription factors. A maize QTL *qKW9* has been reported to encode a pentatricopeptide repeat protein which affects the maternal photosynthates available during grain filling (Huang et al., 2020), while, brassinosteroids are known to play a key role in determining grain size and plant architecture (Zhang et al., 2021). Other gene models code for proteins such as expansin, aldehyde oxidase, N-acetyltransferase, ploygalacturonase, allene oxide cyclase, etc., which are known to play important roles in cell wall loosening, elongation, and cell division (Agrawal et al., 2003; Riemann et al., 2013; Byeon and Back, 2014; Marowa et al., 2016; Lee and Back, 2017; Cao et al., 2021; Shi et al., 2021), which may therefore be associated with an increased grain length. Furthermore, fine-mapping and functional validation is required to pin-point the gene underlying this important QTL.

The KER is one of the important traits of Basmati rice which determines the degree of linear elongation after cooking. This trait is proportional to other economically important cooking quality traits such as volume expansion and water uptake. In the current study, we have identified a novel QTL *viz., qKER2.1* which has a large effect on KER in Basmati rice.

In all, the Basmati rice grain length is mainly governed by three loci, namely *GS3, qGL7.1,* and *qMRL3.2*. However, the cooked grain length is mainly governed by four loci, *viz., GS3, qGL7.1, qCKL3.2,* and *qCKL4.1*. Therefore, targeting these loci through marker-assisted breeding can help in developing Basmati rice genotypes with extra-long slender grains and exceptional cooking quality traits.

## Conclusion

The present study identified novel QTLs governing grain dimension traits in a RIL population generated by crossing a short grain aromatic rice landrace, Sonasal and Pusa Basmati 1121, a Basmati rice variety possessing extra-long slender grains. Two previously reported QTLs and two novel QTLs identified in the current study were identified to influence the exceptional milled rice length and cooked kernel length in Basmati rice. One of the novel QTLs was bracketed to a genomic region of 0.78 Mb which possesses putative candidate genes. However, further fine mapping and map-based cloning is required to precisely identify the candidate genes governing the trait. Furthermore, the markers flanking the QTLs identified can be utilized in the marker-assisted breeding to develop Basmati rice varieties with extra-long slender grains and high kernel elongation upon cooking

## Data Availability

The original contributions presented in the study are included in the article/Supplementary Material; further inquiries can be directed to the corresponding author.
